# PLA/BC/lard nanofiber composites as next-generation burn wound dressings

**DOI:** 10.1039/d6ra00477f

**Published:** 2026-03-16

**Authors:** Tubanur Avci, Natavan Ismayilova, Omer Kocal, Eren Yolgosteren, Zehra Kanli, Ismail Ates, Banu Aydin, Savas Evran, Oguzhan Gunduz, Canan Dogan

**Affiliations:** a Department of Metallurgical and Materials Engineering, Faculty of Technology, Marmara University Istanbul 34722 Turkey canan.dogan@marmara.edu.tr; b Department of Biophysics, Faculty of Medicine, Marmara University Istanbul 34854 Turkey; c Chemical Engineering Department, Engineering Faculty, Istanbul University-Cerrahpasa 34320 Istanbul Turkey; d Marmara University, Faculty of Applied Sciences, Department of Jewelry and Jewelry Design 34865 Istanbul Turkey

## Abstract

This study describes the development of electrospun nanofibers composed of polylactic acid (PLA), bacterial cellulose (BC), and lard for wound dressing applications. PLA provided structural stability, BC enhanced hydrophilicity and mechanical reinforcement, while lard contributed elasticity and biofunctionality. Morphological analysis showed uniform, bead-free fibers; BC incorporation reduced fiber diameter, while lard incorporation increased fiber thickness due to its plasticizing effect on the polymer matrix. Mechanical tests revealed that PLA/BC composites exhibited higher tensile strength, while PLA/BC/lard composites showed greater elongation at break and elasticity, making them suitable for dynamic anatomical regions; numerical analyses confirmed the high reliability of the structural modeling by showing a maximum deviation of 1.57% between experimental and theoretical stress values. Biological evaluations with human dermal fibroblasts confirmed high biocompatibility. PLA/BC/lard nanofibers promoted cell viability, accelerated wound closure, and exhibited a reduction trend in pro-inflammatory cytokines (IL-6 and IL-17) compared to PLA/BC controls, suggesting a non-significant trend of lard incorporation. In conclusion, the synergistic integration of PLA, BC, and lard produced multifunctional nanofibers with balanced mechanical properties, biodegradability, and biological activity. To the best of our knowledge, this is the first study reporting lard-containing PLA/BC nanofibers, highlighting their novelty and potential as next-generation wound dressings with improved early healing-related responses and a non-significant downward trend in pro-inflammatory cytokine levels.

## Abstract 2

Novel PLA/BC/lard nanofibers combine structural strength with lipid-mediated elasticity and bioactivity, creating a mechanically adaptive dressing for accelerated wound healing.

## Graphical abstract

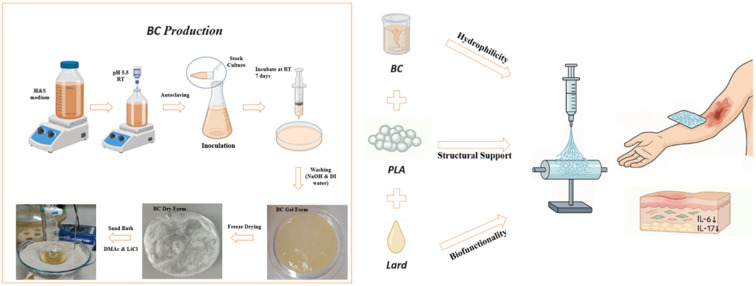

## 1. Introduction

Wound healing is a complex and dynamic biological process encompassing hemostasis, inflammation, proliferation, and remodeling stages, which requires an optimal environment to promote tissue regeneration.^1^ Modern wound dressings are designed not only to protect the wound area but also to actively contribute to the healing process through moisture regulation, oxygen permeability, and the delivery of bioactive compounds. Emerging strategies have further expanded this functionality by introducing mechanically active dressings (MADs) that can autonomously contract to physically accelerate wound closure.^2^ Concurrently, advanced nanotechnology has enabled the development of multifunctional nanoplatforms such as tetrahedral framework nucleic acids (tFNAs), which facilitate cell proliferation and migration while regulating inflammation, alongside coordination polymer particles (CPPs) that offer synergistic therapies.^3–5^ Moreover, novel textile engineering approaches providing ‘attack and defense’ antimicrobial capabilities have shown promise in preventing infection.^6^ Electrospun nanofibers have garnered increasing interest in this context due to their high surface area-to-volume ratio, porosity, and ability to mimic the extracellular matrix (ECM), serving as effective delivery platforms for bioactive agents to accelerate chronic wound healing.^7–9^

Burn wounds involve coagulative necrosis and a heterogeneous lesion that may progress over time, often described as three zones (coagulation, stasis, hyperemia). Microvascular dysfunction and capillary leakage increase edema/exudate and reduce oxygen-nutrient delivery, while eschar formation can delay re-epithelialization and increase infection risk.^10,11^ At the molecular level, DAMP-driven inflammation elevates cytokines (*e.g.*, IL-1β, IL-6, TNF-α) and sustains protease/ROS activity, impairing ECM integrity and keratinocyte/fibroblast migration.^11,12^ Dysregulated remodeling (often TGF-β-associated) may lead to hypertrophic scarring and contracture.^13^ Burn wounds are characterized by disruption of epidermal–dermal barrier integrity, which increases exudative fluid loss and susceptibility to infection, and may promote progressive wound deepening when local control is inadequate. In deeper partial-thickness burns, delayed re-epithelialization (often beyond 3 weeks) is associated with substantially higher hypertrophic scarring risk, underscoring the need for dressings that balance exudate/moisture control with support for timely wound closure.^14^ Accordingly, burn dressings should be explicitly designed to address burn-specific needs—barrier restoration/TEWL control while remaining breathable, moisture-exudate management under variable loads, infection-risk reduction, and inflammatory modulation—to support timely re-epithelialization and reduce fibrosis/hypertrophic scarring.^15,16^

Polylactic acid (PLA), a biodegradable thermoplastic polyester derived from renewable sources, is widely used in biomedical applications due to its biocompatibility, mechanical strength, and processability through electrospinning.^17^ However, pure PLA-based fibers often suffer from brittleness and hydrophobicity, which can limit their performance in moist wound environments. To overcome these limitations, composite formulations incorporating hydrophilic and bioactive components have been investigated.

Bacterial cellulose (BC) is a natural polymer produced by microorganisms such as *Gluconacetobacter xylinus*, known for its high purity, water-holding capacity, and excellent mechanical properties. Its incorporation into electrospun PLA fibers has been shown to improve moisture retention, fiber flexibility, and biocompatibility.^18^ Recent studies have demonstrated that electrospun PLA/BC composite membranes exhibit enhanced structural integrity and wet stability in applications such as air filtration, confirming the synergistic interaction between the hydrophilic BC nanofibers and PLA matrix.^19,20^

Lard, or rendered pig fat, is a natural lipid source that has historically been used in traditional ointments and salves due to its emollient, occlusive, and skin-softening properties.^21^ From a compositional standpoint, lard is rich in triglycerides and fatty acids, particularly oleic acid (∼40–60%) and palmitic acid (∼25–28%), which are critical components of the intercellular lipid matrix responsible for restoring skin barrier integrity and limiting excessive water loss.^22–24^

Traditionally, lard has been regarded as a passive occlusive agent capable of reducing transepidermal water loss (TEWL) by forming a semi-permeable lipid layer on the skin surface.^24^ However, recent biochemical investigations have redefined lard as a biologically active lipid matrix rather than merely an inert vehicle. In particular, Capó *et al.* demonstrated that the thermal rendering and hydroalcoholic extraction of lard yields several bioactive lipid mediators, including 5-dodecanolide and Resolvin D1, which exhibit potent anti-inflammatory activity. These compounds have been shown to suppress the production of tumor necrosis factor-alpha (TNF-α), a key pro-inflammatory cytokine, and to modulate neutrophil activity in both *in vivo* inflammation models and lipopolysaccharide (LPS)-stimulated immune cells. This characteristic, combined with previously reported pro-healing and anti-inflammatory effects in experimental wound models, indicates that lard could be particularly beneficial in managing wounds compromised by barrier disruption, such as extensive excisional wounds or potentially burns, where moisture retention is critical for healing.^22–24^

Supporting these mechanistic findings, an *in vivo* comparative study using rat incision wound models reported that topically applied pig lard promoted fibroblast activity and epithelial regeneration, yielding wound-healing outcomes comparable to, and in some parameters superior to, mupirocin ointment.^23^ Collectively, these findings suggest that lard represents a multifunctional lipid material combining physical barrier restoration with bioactive immunomodulatory effects. Building on this emerging evidence, the present study explores the integration of lard into an advanced wound dressing system, aiming to translate its newly identified bioactive properties into a modern biomaterial context. The synergistic interaction between hydrophilic BC and lipid-rich lard is anticipated to regulate surface wettability and water vapor permeability while establishing a bioactive fibrous interface that promotes fibroblast adhesion, proliferation, and migration. Moreover, the addition of lipid-based components into wound dressings has emerged as a novel strategy for enhancing skin regeneration and maintaining a moist wound environment.

Although numerous studies have investigated PLA-based or PLA/BC-based nanofibers for wound healing, limited attention has been given to the incorporation of lipidic components—particularly natural fats such as lard—into electrospun systems to simultaneously enhance flexibility, moisture regulation, and cellular interactions. Existing literature primarily focuses on polymeric or polysaccharide-based composites, leaving the potential of lipid–polymer hybrid structures largely unexplored.

Electrospun PLA/BC/lard nanofibers provide an ECM-like fibrillar microenvironment by forming a nano/micro-fibrous network with high surface area and interconnected porosity, which can facilitate cell attachment and migration while enabling fluid/nutrient transport through the scaffold. Here, the primary ECM features mimicked are the fibrous architecture, porous structure, and compliant support typical of soft-tissue matrices.^25^ The ECM-mimicking behavior is further supported by BC-driven water-holding capacity and hydrophilicity, which are widely reported as key advantages of bacterial cellulose in wound-dressing applications.^26^ In addition, the lipidic lard phase may contribute to flexibility by acting as a plasticizer-like additive, consistent with reports using animal lard-derived (pork fat) oils as plasticizers and the established mechanism by which plasticizers increase polymer-chain mobility and material flexibility.^27^ The material is tuned by adjusting BC and lard contents and by controlling electrospinning parameters that influence fiber morphology and porosity.^28^

The development of multifunctional nanofibers integrating PLA, BC, and lard offers a promising strategy for burn wound management by addressing the limitations of conventional dressings. In this study, we aimed to develop such a nanofiber to bridge the gap in current therapies. The resulting PLA/BC/lard nanofibers were designed to harness the mechanical reinforcement and water retention capabilities of BC, the processability of PLA, and the plasticizing, anti-inflammatory effects of lard. This novel lipid–polymer composite system addresses brittleness and hydrophobicity while enhancing fibroblast proliferation and wound closure. Therefore, these electrospun nanofibers represent a next-generation approach uniting biocompatibility, mechanical adaptability, and therapeutic functionality.

## 2. Materials and methods

### 2.1. Materials

Lard was obtained from a local livestock producer. PLA (*M*_n_ = 13 000, 2003D) was supplied by NatureWorks LLC (USA). Dimethyl sulfoxide (DMSO, 200 µL, ≥99.9%, molecular biology grade) was purchased from Merck (Germany). Chloroform (analytical grade, ≥99.8%) was obtained from Sigma-Aldrich (St. Louis, MO, USA). Glucose (*M*_w_ = 180.16 g mol^−1^), citric acid (*M*_w_ = 192.12 g mol^−1^), and disodium hydrogen phosphate (*M*_w_ = 141.96 g mol^−1^) were also purchased from Merck (Darmstadt, Germany). Casein peptone Type I and yeast extract were obtained from Neogen (Lexington, KY, USA). Glacial acetic acid (AA, *M*_w_ = 60.05 g mol^−1^, ≥100%), dimethylacetamide (DMAc, *M*_w_ = 87.12 g mol^−1^, ≥99.0%), and lithium chloride (LiCl, *M*_w_ = 42.39 g mol^−1^) were supplied by ISOLAB (Windsor, ON, Canada).

### 2.2. Bacterial cellulose production

**Fig. 1:**
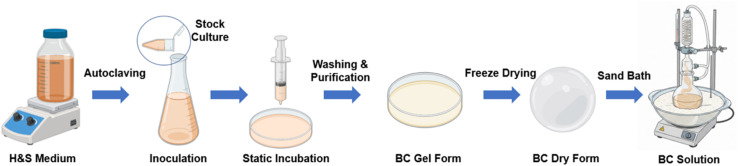
Schematic representation of the BC production and dissolution process.

Gluconacetobacter xylinus (ATCC 23767, Virginia, USA) was used for the production of BC pellicles. The bacteria were cultivated in Hestrin and Schramm (HS) medium containing 2% (w/v) glucose, 0.5% (w/v) yeast extract, 0.5% (w/v) peptone, 0.27% (w/v) disodium hydrogen phosphate, and 0.115% (w/v) citric acid in 1 L of distilled water. The pH of the medium was adjusted to 5.5 using acetic acid (AA), which has been reported as optimal for cellulose production. The medium was then sterilized by autoclaving at 121 °C for 20 minutes and allowed to cool to room temperature (RT). For preculturing, the cells were initially grown in test tubes and subsequently transferred into 100 mL Erlenmeyer flasks containing 40 mL of HS medium. The flasks were incubated statically at RT for 3 days. Once a thin cellulose pellicle formed on the surface, 1% of the culture (including cells and medium) was transferred first to a fresh flask and then to Petri dishes, where static incubation continued at RT for an additional 7 days. The resulting BC pellicles were purified by immersion in 0.1 M sodium hydroxide at RT for 24 hours to remove bacterial cells and residual medium components. The pellicles were then thoroughly washed with deionized water overnight to neutralize the solution. Subsequently, they were sterilized again by autoclaving and stored at −20 °C until further use. Prior to application, the frozen pellicles were thawed and freeze-dried. For the dissolution process, a 100 : 1 (v/v) mixture of *N*,*N*-dimethylacetamide (DMAc) and BC was stirred and heated at 170 °C for 1 hour using a sand bath equipped with a condenser system. Subsequently, 0.4% lithium chloride (LiCl) was added, and the solution was stirred at 100 °C for an additional hour. The final step involved stirring at RT for 24 hours, yielding a homogeneous, viscous solution suitable for solvent-based processing. As illustrated in Fig. 1, the schematic diagram presents the complete process of BC production and its subsequent dissolution *via* sand bath heating, ultimately yielding a uniform solution suitable for electrospinning.^29^

### 2.3. Electrospinning process

To prepare the electrospinning solutions, PLA (10% w/v) and lard (1% w/v) were dissolved in a chloroform/DMSO binary solvent (9 : 1 v/v) under magnetic stirring at 25 ± 2 °C for 4 h (or until complete dissolution). In parallel, BC was prepared at 2% (w/v) in DMAc and stirred at 25 ± 2 °C for 12 h to obtain a visually homogeneous dispersion/solution. The PLA/lard and BC solutions were then combined at a 3 : 1 (v/v) ratio and stirred at 25 ± 2 °C for 12 h (overnight). During all preparation steps, the solutions were kept in closed containers to minimize solvent evaporation. The final mixture was loaded into a 10 mL syringe fitted with a 20-gauge stainless steel needle. Electrospinning was conducted at ambient conditions (25 ± 2 °C, relative humidity 40–50%) using an applied voltage of 22 kV, a tip-to-collector distance of 15 cm, and a flow rate of 1 mL h^−1^. All electrospinning procedures were carried out at ambient temperature. The obtained nanofibers were collected on non-stick paper and stored at room temperature for subsequent characterization analyses.

### 2.4. Ethics statement

This study was conducted exclusively *in vitro* and did not involve human participants or animal experimentation. The human dermal fibroblast (HDF) cell lines were obtained from a commercial repository (ATCC, PCS-201-012), and the bacterial cellulose was produced using *Gluconacetobacter xylinus* (ATCC 23767). Consequently, specific ethical approval or informed consent was not required for this study.

### 2.5. Scanning electron microscopy (SEM)

The morphological structure of the produced nanofibers was examined using a scanning electron microscope (SEM, ZEISS, Jena, Germany). Prior to imaging, the samples were coated with a thin gold layer under vacuum for 120 s *via* sputtering to prevent electrostatic charging and improve image quality. Fiber diameters were measured from SEM images using ImageJ software (v1.47, NIH, Bethesda, MD, USA), with at least 100 measurements taken from different regions of each sample for statistical accuracy.

### 2.6. Fourier transform infrared (FTIR) spectroscopy

FTIR analysis was carried out to determine the chemical composition and functional groups of the nanofibers. Spectra were recorded using an FT/IR-4700 spectrometer (JASCO Corporation, Tokyo, Japan) in the range of 4000–400 cm^−1^, at a resolution of 4 cm^−1^ with 32 scans. Measurements were performed for neat PLA, BC, lard, and the PLA/BC/lard composites, and characteristic absorption peaks were compared to assess possible intermolecular interactions.

### 2.7. Differential scanning calorimetry (DSC)

Thermal properties were analyzed using a differential scanning calorimetry (DSC) system (DSC-60 Plus instrument, Shimadzu Corporation, Kyoto, Japan). Approximately 2–3 mg of each sample was placed in aluminum pans and heated from 25 °C to 300 °C at a rate of 10 °C min^−1^. The glass transition temperature (*T*_g_) was 63.60 °C for PLA/BC nanofibers and 49.74 °C for PLA/BC/lard nanofibers, indicating the plasticizing effect of lard. The melting temperature (*T*_m_) was approximately 154 °C for both fiber types.

### 2.8. Tensile test

Mechanical properties were determined using a Shimadzu EZ-LX tensile tester (Shimadzu Corporation, Kyoto, Japan). Rectangular strips (10 mm × 50 mm) were tested at a crosshead speed of 5 mm min^−1^ with a preload of 0.1 N. Each test was performed in triplicate to ensure statistical reliability.

#### 2.8.1. Numerical analysis

The stress behaviors of PLA, PLA/BC, and PLA/BC/lard nanofiber composites were numerically calculated using finite element method and the numerical modelling of the composites was carried out utilizing ANSYS WORKBENCH software (Canonsburg, PA, USA). Numerical analyses were implemented for von-Mises and normal stresses and each composite was modelled under fixed and displacement boundary conditions. *X*, *Y*, and *Z* components for the fixed boundary condition were used to be 0 mm, whereas *X* and *Z* components in the displacement boundary condition were determined as 0 mm. The tensile force was calculated along the *Y*-axis and the *Y* component was evaluated freely. Displacement and fixed boundary conditions were respectively applied to the top and bottom surfaces of the composite, while tensile force in *Y*-axis was applied only to the top surface. In numerical modeling, composite dimensions were evaluated according to the dimensions used in experimental analyses. In the finite element modeling, the element size for the mesh analyses was evaluated as 0.1 mm.

### 2.9. Swelling–degradation test

Swelling and degradation tests were carried out on all nanofibrous patch groups to evaluate water absorption capacity and mass loss over time. For the swelling analysis, three equally weighted nanofiber patch specimens from each group were placed in 1 mL Eppendorf tubes containing phosphate-buffered saline (PBS, pH 7.4). The tubes were incubated in a thermal shaker (BIOSAN TS-100) at 37 °C for 24 h. After incubation, the samples were removed, gently blotted, and weighed (*W*_w_) using an electronic balance. The swelling ratio was calculated according to eqn (1):^29^

1 *S* = (*W*_w_ − *W*_d_)/*W*_d_ × 100

For the degradation test, nanofibrous patches were cut into equal pieces, weighed (*W*_0_), and incubated in 1 mL PBS at 37 °C for 24 h in an Eppendorf thermal shaker. Following incubation, the PBS was removed, and the nanofiber samples were transferred back into empty Eppendorf tubes. The tubes were then dried at 37 °C for 24 h with open lids. After drying, the final weights (*W*_t_) of the nanofibers were recorded, and the degradation percentage was calculated using eqn (2):

2 *D* = (*W*_0_ − *W*_t_)/*W*_0_ × 100

### 2.10. *In vitro* assessment of cell viability, cytokine response, and wound healing on nanofibers

#### 2.10.1. Determination of cell viability on nanofibers

The biocompatibility of the fabricated nanofibers was evaluated using human dermal fibroblast cells (HDF, PCS-201-012, ATCC, Maryland). Prior to experimentation, PLA/BC and PLA/BC/lard nanofibers constructs were sterilized by exposing both their front and back surfaces to ultraviolet (UV) light for one hour. For cell viability assessment, the MTT Cell Proliferation and Cytotoxicity Assay Kit (E-CK-A341, Elabscience Biotechnology Co., Ltd, Wuhan, China) was employed. HDF cells were prepared in Dulbecco's Modified Eagle Medium (DMEM, Capricorn, Germany) supplemented with 10% fetal bovine serum (FBS, Capricorn, Germany) and 10 000 units per mL penicillin–streptomycin (Capricorn, Germany), and seeded into 96-well plates at a density of 5 × 10^3^ cells per mL. Each experiment was performed in triplicate. Cells were cultured in a humidified incubator at 37 °C with 5% CO_2_. MTT assays were conducted on days 1, 3, and 7 post-seeding. On each specified day, fresh culture medium (100 µL) was added to each well, followed by the addition of 50 µL MTT stock solution. Plates were then incubated at 37 °C for 2.5 hours. Subsequently, the medium was removed, and 150 µL of dimethyl sulfoxide (DMSO, Sigma-Aldrich, St. Louis, MO, USA) was added to each well to dissolve the formed formazan crystals. Cell viability was quantitatively measured spectrophotometrically at 570 nm using a BioTek microplate reader (Santa Clara, CA, USA). Per ISO 10993-5, viability >70% was considered non-cytotoxic in extract-based *in vitro* cytotoxicity assessment.

#### 2.10.2 Wound healing assay (scratch assay)

HDF cells were seeded in 24-well plates at a density of 2 × 10^4^ cells per well and allowed to reach 90–100% confluence. A linear scratch was created in the cell monolayer using a sterile 200 µL pipette tip (0 h). Wells were gently washed with PBS, and cells were cultured under three conditions: control (HDF cells only), PLA/BC and PLA/BC/lard nanofibers. The same regions were imaged at 0 h and 24 h using phase-contrast microscopy.^30^ Images were analyzed with ImageJ software, and the percentage of wound closure was calculated wound closure (%) = (area at 0 h − area at *t* h)/(area at 0 h) × 100.^31^ Data were expressed as mean ± SEM. Statistical differences between groups were evaluated by one-way ANOVA followed by Tukey's multiple comparisons test, with *p* < 0.05 considered statistically significant.

#### 2.10.3. IL-6 and IL-17 cytokine assay on nanofibers

HDF cells cultured on the respective nanofibers were analyzed for cytokine levels using ELISA. Human IL-6 and IL-17 ELISA kits (BT-Lab, Hangzhou, China) were employed according to the manufacturer's instructions. Briefly, cell culture supernatants were collected at the designated time points, added to pre-coated 96-well plates, and incubated with the provided detection antibodies. After washing steps, substrate solution was added, and absorbance was measured at 450 nm using a microplate reader (BioTek, Winooski, VT, USA). Cytokine concentrations were calculated based on standard curves provided in the kits. Cytokine measurements in this study were performed under basal culture conditions without exogenous inflammatory priming (TNF-α or IL-1β).

#### 2.10.4. Statistical analysis

All experiments were conducted in triplicate (*n* = 3), and data are presented as mean ± SEM. Statistical analyses were performed using GraphPad Prism 8.0.2 (GraphPad Software, San Diego, CA, USA). For MTT data, two-way ANOVA followed by Tukey's multiple-comparisons *post hoc* test was used. For ELISA (IL-6 and IL-17) and wound-healing assays, one-way ANOVA followed by Tukey's multiple-comparisons post hoc test was applied. For swelling data, two-way ANOVA (material × time) followed by Sidak's multiple-comparisons test was used. For PLA/BC/lard degradation data, one-way ANOVA followed by Dunnett's multiple-comparisons test (*vs.* Day 1) was used. A *p* value <0.05 was considered statistically significant.

## 3. Results and discussion

### 3.1. Scanning electron microscopy (SEM)

**Fig. 2:**
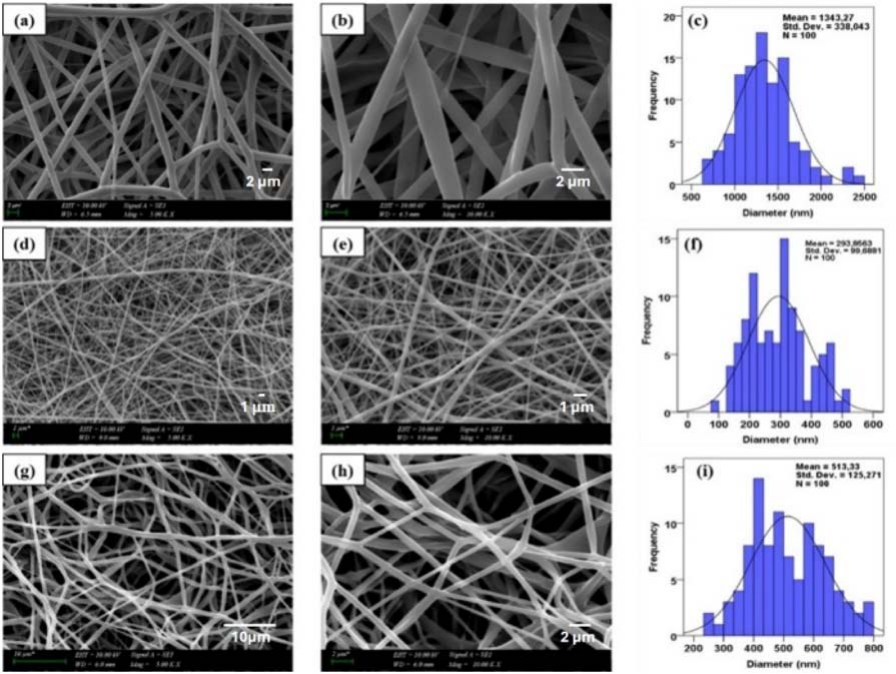
SEM images and diameter distributions of PLA, PLA/BC and PLA/BC/lard nanofibers: (a) and (b) PLA nanofibers at ×5000 and ×10 000 magnifications, respectively; (c) diameter distribution of PLA nanofibers; (d) and (e) PLA/BC nanofibers at ×5000 and ×10 000 magnifications, respectively; (f) diameter distribution of PLA/BC nanofibers; (g) and (h) PLA/BC/lard nanofibers at ×5000 and ×10 000 magnifications, respectively; (i) diameter distribution of PLA/BC/lard nanofibers.

The SEM images and corresponding histograms of fiber diameter distributions for PLA/BC and PLA/BC/lard nanofibers are shown in Fig. 2. Examining the SEM images and diameter distribution histograms, it was determined that the pure PLA nanofibers had an average diameter of 1343 ± 338 nm. In PLA/BC nanofibers, the diameter values decreased significantly with the addition of BC, reaching an average of 293 ± 99 nm. This is due to the hydrophilic nature of BC, which increases jet stability during electrospinning, resulting in the formation of thinner and more homogeneous fibers. Similarly, the literature reports that fibers become thinner and fiber morphology improves in PLA/BC systems.^32,33^

The addition of lard to the PLA/BC system increased the fiber diameter to 513 ± 125 nm. This increase is thought to be due to lard increasing the viscosity of the polymer solution and affecting jet thickness. However, the fibers in all formulations were observed to be uniform, bead-free, and have a homogeneous morphology. Similarly, it is stated in the literature that natural additives affect the diameter distribution in PLA-based fibers but maintain the homogeneity of the fiber morphology.^34^

### 3.2. FTIR analysis

**Fig. 3:**
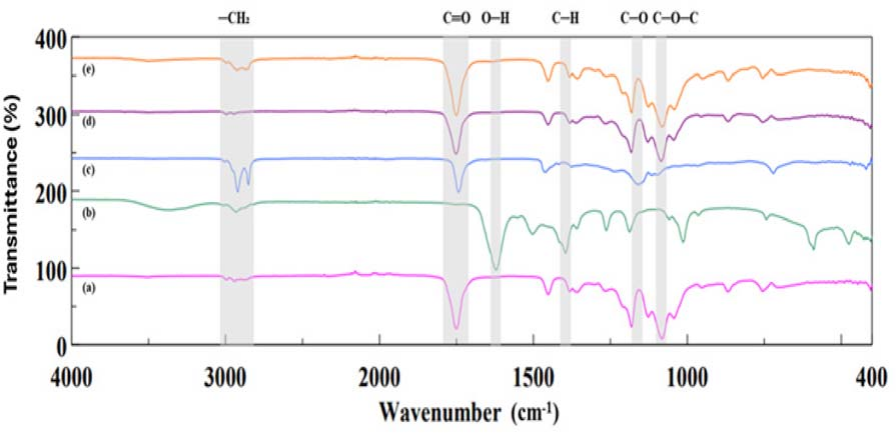
FTIR spectra of (a) PLA, (b) BC, (c) lard, (d) PLA/BC composite, and (e) PLA/BC/lard composite.

In this study, FTIR spectroscopy was performed to investigate the functional groups and molecular interactions within neat PLA, BC, lard, and their composites (Fig. 3).

The FTIR spectrum of neat PLA exhibited a strong absorption band at 1749 cm^−1^, corresponding to the stretching vibration of the C=O group in the polymer backbone. Additional characteristic bands were observed at 1180–1080 cm^−1^, assigned to C–O–C stretching vibrations, and at 2945–2995 cm^−1^, representing the symmetric and asymmetric stretching vibrations of –CH_3_ and –CH_2_ groups^35^

The BC spectrum displayed a broad O–H stretching band in the 3300–3400 cm^−1^ region, indicative of extensive hydrogen bonding and the hydrophilic nature of cellulose. A peak near 2900 cm^−1^ was attributed to aliphatic –CH_2_ stretching vibrations. Sharp absorption bands between 1050–1030 cm^−1^ corresponded to C–O and C–O–C stretching vibrations. Furthermore, a band at 1640 cm^−1^ was associated with O–H bending from absorbed water molecules, while the peak at 1375 cm^−1^ reflected –CH bending vibrations linked to the crystalline domains of cellulose.^36^

The FTIR spectrum of lard showed a prominent carbonyl band around 1745 cm^−1^, corresponding to ester C=O stretching vibrations typical of triglyceride structures.^29^ Strong aliphatic C–H stretching bands were also observed at 2850 cm^−1^ for symmetric and at 2920 cm^−1^ for asymmetric stretching, reflecting the saturated fatty acid chains in lard.^37^ Additionally, weaker bands in the 1160–1090 cm^−1^ range were attributed to C–O stretching vibrations in ester linkages.^38^

The PLA/BC composite spectrum retained both the characteristic 1750 cm^−1^ carbonyl peak originating from PLA and the broad O–H band around 3300 cm^−1^ attributed to BC. The preservation of peak positions without significant shifts or the appearance of new peaks suggests that PLA and BC interact primarily through physical mixing rather than covalent bonding. The presence of BC-related bands at 1640 and 1375 cm^−1^ further indicates the structural integrity of cellulose within the composite matrix.^39^

The FTIR spectra of the PLA/BC/lard composite exhibited distinct retention of characteristic absorption bands from each component. The ester carbonyl band at 1750 cm^−1^, attributed to both PLA and lard, was clearly observed along with intense aliphatic C–H stretching bands between 2850 and 2950 cm^−1^, originating from lard. Additionally, a broad O–H stretching band centered around 3300 cm^−1^ confirmed the presence of BC. The enhanced intensity of the C–H bands indicated effective incorporation of lard into the composite.

The FTIR spectrum of the PLA/BC/lard composite revealed distinct vibrational bands attributable to each individual component, indicating that their chemical structures remained intact following the fabrication process. This observation confirms that PLA, bacterial cellulose, and lard were successfully incorporated into the composite without undergoing significant chemical modification, thereby preserving their functional contributions within the matrix.

### 3.3. Differential scanning calorimetry (DSC)

**Fig. 4:**
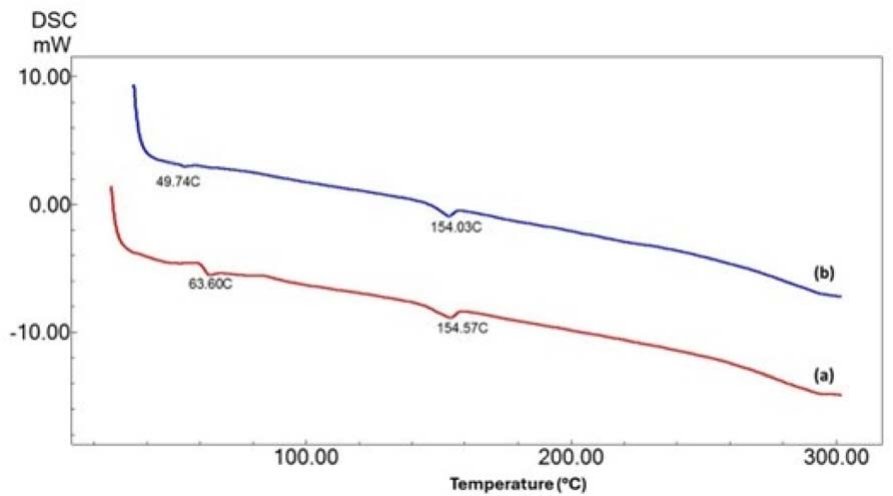
Differential scanning calorimetry curves of: (a) PLA/BC fiber, (b) PLA/BC/lard fiber.

DSC curves for PLA/BC and PLA/BC/lard fibers are presented in Fig. 4. The glass transition temperature (*T*_g_) was determined as 63.60 °C for pure PLA/BC fibers and 49.74 °C for PLA/BC/lard fibers containing 1% lard. The addition of lard increased polymer chain mobility, leading to a decrease in *T*_g_, demonstrating the additive's plasticizing effect.

In the literature, the *T*_g_ value of PLA is generally reported to be around 60 °C and its melting temperature (*T*_m_) is between 150–155 °C.^40^ While the *T*_g_ of bacterial cellulose has been reported as ∼62.7 °C in some composite systems, a pure BC film was found to exhibit a *T*_g_ of −30.3 °C and a *T*_m_ of 85.6 °C.^41^ Lard, on the other hand, is not a polymeric structure, but its *T*_g_ varies between −6 and −10 °C and its *T*_m_ varies between 39–48 °C.^38^

Considering these literature findings, the decrease in *T*_g_ observed in our study suggests that lard weakens interchain interactions by increasing segmental mobility in the PLA matrix, thus leading to lower glass transition temperatures. Similarly, Mena-Prado *et al.* (2025) reported that fatty acid esters reduce *T*_g_ by increasing flexibility in PLA. It has also been reported that oil-based additives increase free volume, facilitating chain mobility, and thus have a reducing effect on *T*_g_.^42^

When examining the melting temperatures, the *T*_m_ value for both fiber samples was approximately 154 °C, and no significant effect of the lard additive on this parameter was observed. This may be due to the limited effect on the crystalline structure due to the low additive rate (1%). Indeed, Mena-Prado *et al.* (2025) reported that Tm remained largely constant with fatty acid additives, while Orisekeh *et al.* (2025) reported only a few degrees of *T*_m_ decrease even at higher additive rates. These findings support the idea that low-rate oil additives do not disrupt the crystalline phase of the PLA matrix and do not cause a significant change in melting temperature.^42,43^

These results indicate that the lard addition reduces the glass transition temperature by imparting flexibility to the PLA/BC matrix, but does not cause significant deterioration in the crystalline structure. Lowering *T*_g_ makes the wound dressing material more flexible and compatible with the body, while maintaining a constant *T*_m_ offers advantages in terms of thermal stability. In this context, the resulting composite fibers are considered to have potential for wound dressing applications due to their mechanical flexibility and thermal resistance.

### 3.4. Swelling–degradation tests

**Fig. 5:**
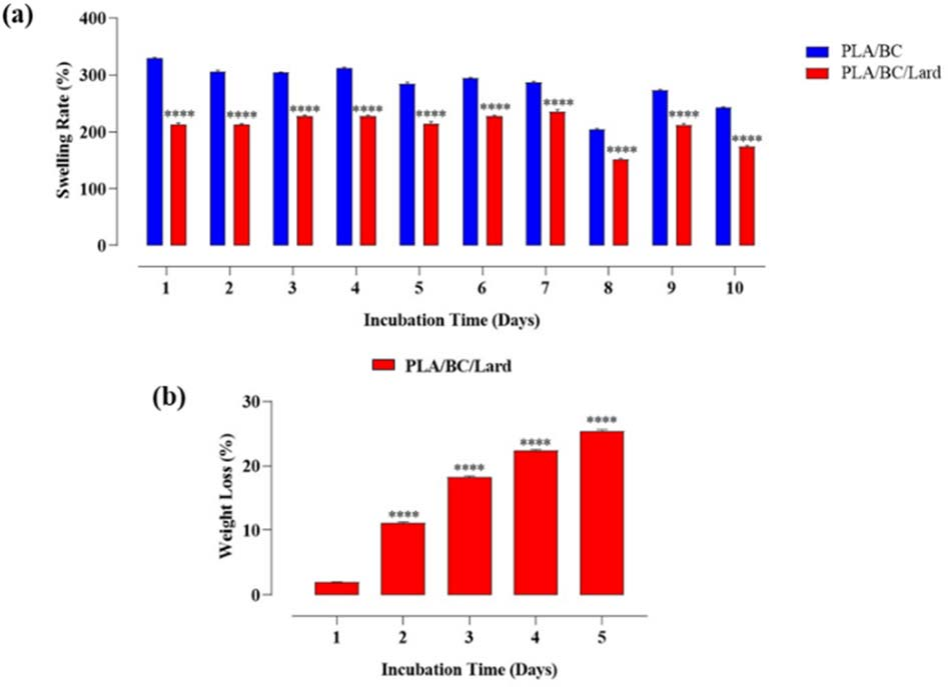
(a) Swelling behavior of PLA/BC and PLA/BC/lard composites over 10 days in PBS (pH 7.4). Statistical analysis was performed using two-way ANOVA (material × time) followed by Sidak's multiple-comparisons test; symbols indicate PLA/BC *vs.* PLA/BC/lard differences at the corresponding day (b) degradation behavior (mass loss) of PLA/BC/lard over 5 days in PBS (pH 7.4). Data are presented as mean ± SEM (*n* = 3). One-way ANOVA followed by Dunnett's multiple-comparisons test (*vs.* Day 1) was used. *p < 0.05, **p < 0.01, ***p < 0.001, ****p < 0.0001; ns, not significant.

Swelling and degradation behaviors are critical indicators of the hydrophilicity, porosity, and interaction of polymer matrices with aqueous environments, directly affecting their mechanical performance and biocompatibility, especially in biomedical applications such as wound dressings, scaffolds, and drug delivery systems^44^ In this study, the swelling and degradation rates of PLA/BC and PLA/BC/lard nanocomposites were systematically investigated during an incubation period of 5 and 10 days under physiological conditions (PBS, pH 7.4), and the results are shown in Fig. 5.

As shown in Fig. 5a, the PLA/BC group initially exhibited a significantly higher swelling ratio (∼330%) compared to PLA/BC/lard (∼220%) on Day 1. This trend continued consistently throughout the incubation period, with both groups showing a gradual decrease in swelling and reaching a plateau around Day 10, where PLA/BC decreased to approximately 260% and PLA/BC/lard to approximately 180%. The superior swelling capacity of PLA/BC can be attributed to the highly hydrophilic and nanofibrous network structure of BC, which enables extensive water uptake due to its large surface area and hydroxyl-rich molecular backbone.^45^

In contrast, the incorporation of lard (PLA/BC/lard group) significantly reduced the swelling capacity of the composites. This reduction is explained by the hydrophobic nature of lard, which limits water penetration and restricts polymer chain mobility, thereby reducing water absorption capacity. Such hydrophobicity-induced suppression of swelling has also been consistently reported in the literature for natural fat or wax-based inclusions within hydrophilic biopolymers.^46^ Moreover, lard molecules may occupy void spaces or inter-fiber gaps within the BC matrix, thereby limiting the swelling-active surface area.

Interestingly, both groups exhibited a notable decrease in swelling between Days 7 and 8, which may be due to structural relaxation, partial degradation, or saturation effects, leading to reduced water affinity. Similar phenomena have also been observed in other biopolymer hydrogels, where extended immersion times result in structural fatigue or hydrolytic changes that compromise further water uptake.^47^

In summary, the PLA/BC nanocomposite demonstrated superior swelling behavior due to the intrinsic hydrophilicity of BC, whereas PLA/BC/lard exhibited diminished swelling capacity as a result of the hydrophobic influence of lard.

In the biodegradation experiments of PLA/BC composite nanofibers, no significant mass loss was detected after short-term incubation. This finding is consistent with reports in the literature on the degradation behavior of BC and PLA. BC possesses a highly crystalline structure due to strong hydrogen bonds between its hydroxyl groups, making it resistant to enzymatic and chemical degradation.^48,49^ Similarly, the degradation of PLA in biological environments generally occurs over months to years, which limits visible mass loss in short-term tests.^50^ Indeed, the BC/PVP electrospun composites reported by Cesur *et al.* (2024) did not show any mass loss in lysozyme solution for 28 days, suggesting that BC based composites offer highly stable structures in the short term.^51^ Therefore, the absence of a degradation graph in our study is attributed to the inherently low degradation tendency of the polymers used under short-term biological conditions.^51^

Fig. 5b presents the degradation behavior of the PLA/BC/lard composite over a five-day incubation period. The results clearly demonstrate that the PLA/BC/lard composite exhibits a time-dependent and progressive degradation profile. On the first incubation day, degradation remained at a minimal level, indicating that the composite structure retained its stability against the surrounding medium. From the second day onward, a marked increase in degradation was observed, reaching approximately 10%. This trend continued steadily, with a significant acceleration noted between Days 3 and 4, ultimately reaching a maximum of around 25% by the fifth day.

The observed degradation pattern can be explained by the synergistic effects of the composite's constituents. PLA is prone to hydrolytic cleavage of ester bonds in aqueous environments, progressively weakening the polymer matrix^52^ BC, although highly crystalline and resistant to direct degradation, provides a porous morphology that facilitates water diffusion into the matrix, thereby accelerating PLA hydrolysis.^53^ In addition, the incorporation of lard introduces a hydrophobic component, which limits initial water uptake. However, regarding the mass loss, the significant reduction observed (∼25%) implies a mechanism other than polymer hydrolysis, given that neat PLA and BC are stable over short periods in PBS. Since FTIR analysis confirmed that lard is physically mixed within the matrix without covalent bonding, the mass loss is attributed to the progressive diffusion and physical detachment of lipid components into the aqueous medium. This leaching effect creates voids within the structure, resulting in the measured weight reduction even in the absence of enzymatic degradation.^54^

PLA-based materials may generate acidic degradation products during hydrolysis, and localized acidification can develop within the matrix under diffusion-limited conditions.^55^ Therefore, lactic acid arising from PLA degradation may influence wound-repair processes; however, the direction and magnitude of this effect depend on local microenvironmental factors, including pH, fluid exchange, and exposure duration. Consistently, prolonged strongly acidic conditions have been reported to negatively affect wound closure and re-epithelialization.^56^ Because the pH microenvironment was not directly mapped in the present study, no causal pH-effect inference is made. Nevertheless, under short-term *in vitro* conditions, no clear adverse trend was observed in cytocompatibility or cell-migration readouts. Time-resolved pH monitoring in burn-relevant models could provide a more direct assessment of this relationship.

As shown in Fig. 5a, PLA/BC exhibited higher swelling ratios than PLA/BC/lard throughout the 10 days incubation period. Two-way ANOVA (material × time) followed by Sidak's post hoc test showed significant between-group differences at the corresponding days (****p < 0.0001). A transient minimum was observed around Day 8 in both groups, followed by a partial rebound at Days 9–10. This pattern may reflect a biphasic behavior involving early network relaxation/rearrangement and later matrix heterogeneity associated with progressive component release/leaching.^57,58^ In Fig. 5b, PLA/BC/lard showed progressive mass loss over time. One-way ANOVA with Dunnett's *post hoc* test (*vs.* Day 1) demonstrated significant increases from Days 2–5 (****p < 0.0001). These findings indicate a time-dependent degradation/release profile under the tested PBS conditions.

### 3.5. Tensile tests

**Fig. 6:**
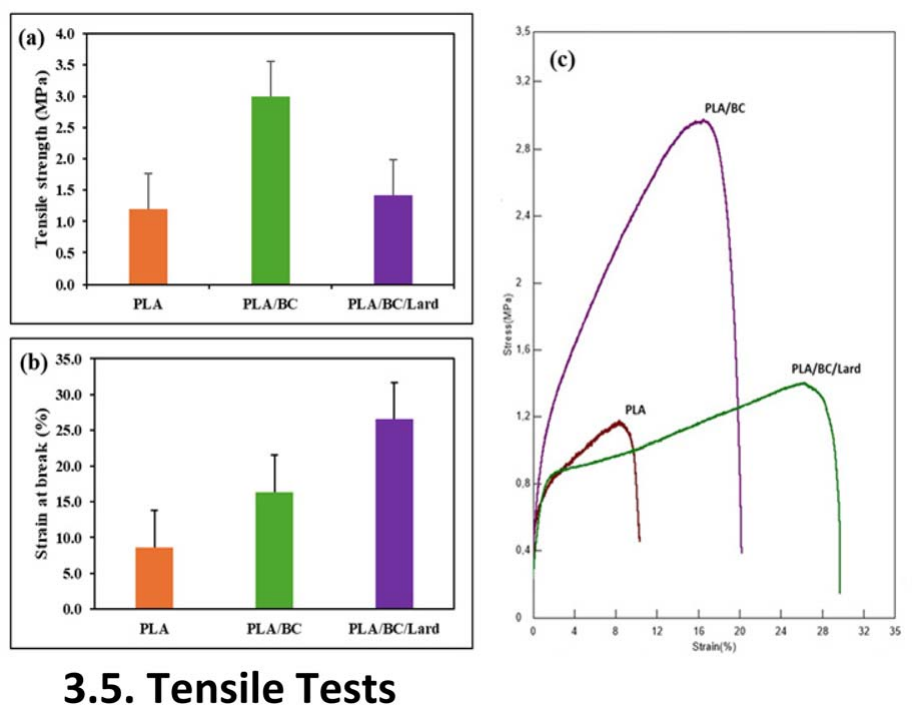
Tensile properties of PLA, PLA/BC, and PLA/BC/lard composites: (a) maximum tensile strength (MPa); (b) elongation at break (%), (c) average stress–strain curves of PLA, PLA/BC, and PLA/BC/lard composites under tensile loading.

The clinical effectiveness of wound dressings largely depends on their mechanical properties, especially in terms of anatomical location, mobility, and external stress exposure.^59^Fig. 6a and b illustrate the tensile strength and elongation at break of neat PLA, PLA/BC, and PLA/BC/lard composites.

As presented in Fig. 6a, neat PLA displayed a relatively low tensile strength of 1.19 ± 0.002 MPa, underscoring its brittle nature and limited mechanical endurance under stress. This restricts its application as a standalone wound dressing, especially in mechanically active or load-bearing areas. However, the incorporation of BC significantly enhanced the material's strength, with the PLA/BC composite reaching 2.99 ± 0.22 MPa. This improvement is primarily attributed to the reinforcing network provided by BC fibers, which facilitate effective stress distribution within the PLA matrix, inhibit crack propagation, and improve structural cohesion.^60^ Accordingly, PLA/BC composites appear suitable for wound dressings intended for relatively immobile anatomical regions requiring mechanical robustness.

The PLA/BC/lard composite exhibited a moderate tensile strength of 1.41 ± 1.13 MPa slightly improved relative to neat PLA but notably lower than PLA/BC. This reduction is likely due to the plasticizing effect of lard, which disrupts inter-chain interactions and compromises the efficiency of load transfer within the matrix.^61^

Fig. 6b illustrates the elongation at break, which reflects the ductile behavior of the materials. Neat PLA showed limited deformability 8.65 ± 1.01%, consistent with its inherently brittle profile. The PLA/BC composite achieved an elongation of 16.35 ± 1.71%, indicating that cellulose addition improves not only strength but also flexibility. Most notably, the PLA/BC/lard composite exhibited a pronounced increase in elongation, reaching 26.49 ± 0.64%, which can be attributed to the plasticizing nature of lard. The reduction in intermolecular forces facilitates greater chain mobility, thereby enabling the material to undergo extensive deformation without rupture.^62^ This increased elasticity is particularly beneficial in dynamic body regions such as joints, where frequent movement demands conformability and resilience. Enhanced flexibility also aids in maintaining continuous contact with the wound site, minimizing detachment and preserving an optimal healing environment.^63^ This capability aligns with the emerging concept of ‘mechanically active dressings (MADs)’, which represents a shift from passive wound coverage to active mechanobiological modulation. As highlighted in recent reviews, dressing materials that possess mechanical adaptability matching human tissues can significantly accelerate wound closure and reduce scarring by optimizing the mechanical microenvironment and influencing mechanotransduction pathways.^2^

Additionally, Fig. 6c presents the average stress–strain curves of PLA, PLA/BC, and PLA/BC/lard composites under tensile loading. These curves clearly illustrate the mechanical responses of the materials.

To validate these experimental findings and evaluate the structural reliability of the composites *via* computational modeling, a numerical analysis was performed. The von-Mises and normal stresses of the nanofiber composites were calculated numerically using the Finite Element Method (FEM) and compared with the experimental stress results. The percentage differences between the theoretical and experimental values were determined to assess the accuracy of the model. The comparative data for numerical and experimental stresses are presented in Table 1.

**Table 1:** Comparison of numerical and experimental stresses

Composites	Units	Experimental analyses	Numerical analyses		% Dif.
		Experimental stress	von-Mises stress	Normal stress	
PLA	MPa	1.0334	1.0172	1.0172	1.57
PLA/BC	MPa	2.9955	2.9887	2.9887	0.23
PLA/BC/lard	MPa	1.4175	1.4094	1.4094	0.57

**Fig. 7:**
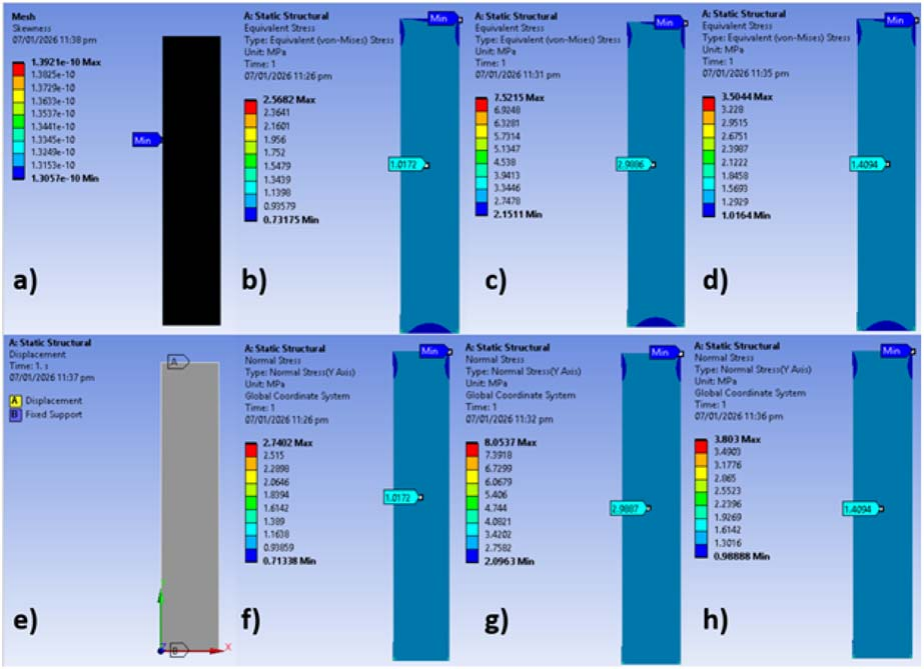
Contour plots; (a) mesh quality, (b) von-Mises stress of PLA composite, (c) von-Mises stress of PLA/BC composite, (d) von-Mises stress of PLA/BC/lard composite, (e) boundary conditions, (f) normal stress of PLA composite, (g) normal stress of PLA/BC composite, (h) normal stress of PLA/BC/lard composite.

As presented in Table 1, the numerical analysis yielded identical values for von-Mises and normal stresses within each composite group. When comparing experimental and numerical data, the maximum deviation was observed in the neat PLA group (1.57%), followed by PLA/BC/lard (0.57%) and PLA/BC (0.23%). These minor discrepancies can be primarily attributed to the geometric differences between the physical samples and the computational models. Experimentally, the nanofibers possess a porous structure with voids, which reduces the effective cross-sectional area capable of bearing the tensile load. In contrast, the numerical models assumed a solid, continuous geometry without pores or defects. This idealization in the finite element analysis likely accounts for the slight variations observed. The resulting stress distributions are visually represented in the contour plots provided in Fig. 7.

Fig. 7a displays mesh quality based on skewness approach and this value was considered as a maximum of 1.4 × 10^−10^. Fig. 7b–d illustrate the von-Mises stresses of PLA, PLA/BC, and PLA/BC/lard composites. The lowest von-Mises stresses were occurred in regions near the boundary conditions, while average stresses were monitored in the middle regions of the composites. Fig. 7e shows the boundary conditions such as displacement and fixed support. Fig. 7f–h demonstrate the normal stresses of PLA, PLA/BC, and PLA/BC/lard composites. Average stresses were detected in the middle regions of the composites in the normal stress analysis.

In conclusion, PLA/BC composites offer enhanced tensile strength, making them favorable for wound dressings applied to stable anatomical sites. In contrast, PLA/BC/lard composites, while mechanically softer, deliver superior elasticity and user comfort—attributes that are crucial for dressings in motion-intensive regions. Despite the slight reduction in tensile performance, the inclusion of lard significantly improves the biomechanical adaptability of the composite, highlighting its potential as a flexible and patient-friendly wound dressing material. Furthermore, numerical results validated these experimental findings, showing a deviation of at most 1.57% between experimental and theoretical stress values.

### 3.6. Cellular responses to nanofibers: viability, cytokine response, and wound closure

#### 3.6.1. *In vitro* cell viability assessment

**Fig. 8:**
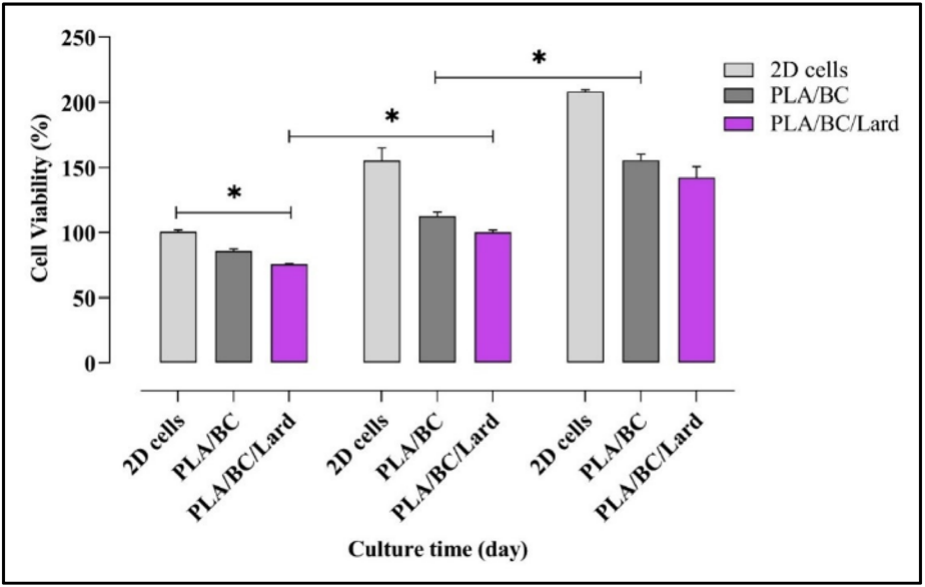
Cell viability of HDF cells was assessed by MTT assay. The biocompatibility of HDF cells incubated with nanofibers was evaluated on day 1, 3 and 7. Data are presented as mean ± SEM (*n* = 3). Statistical analysis was performed using two-way ANOVA followed by Tukey's multiple comparisons test. *p < 0.05, **p < 0.01, ***p < 0.001; ns, not significant (*p* > 0.05).

The biocompatibility of the PLA/BC/lard composite was assessed using the MTT assay on days 1, 3, and 7 after seeding with HDF cells (Fig. 8). The results showed a time-dependent increase in cell viability, indicating progressive cellular adaptation and proliferation throughout the incubation period. On day 1, the PLA/BC/lard group exhibited cell viability above the minimum biocompatibility threshold of 70%, suggesting that the composite initially provides a moderately favorable microenvironment for HDF cell attachment and survival (*p < 0.05). By day 3, cell viability significantly increased (*p < 0.05), reflecting enhanced proliferation and metabolic activity within the nanofiber, which indicates good biocompatibility and cellular adaptation. On day 7, the PLA/BC/lard group reached peak viability (*p < 0.05), confirming robust cellular proliferation and excellent long-term compatibility. This marked increase also supports the composite's potential to sustain cell growth under prolonged culture conditions. According to ISO 10993-5-aligned interpretation for extract-based *in vitro* cytotoxicity assays, cell viability above 70% is generally considered non-cytotoxic. Therefore, the observed viability values support *in vitro* cytocompatibility of the PLA/BC/lard construct under the tested conditions, rather than definitive clinical suitability. Overall, the PLA/BC/lard composite maintained cell viability above the 70% threshold at all evaluated time points, indicating non-cytotoxicity and *in vitro* cytocompatibility under the tested conditions.

#### 3.6.2. Evaluation of wound closure and cell migration on nanofiber

**Fig. 9:**
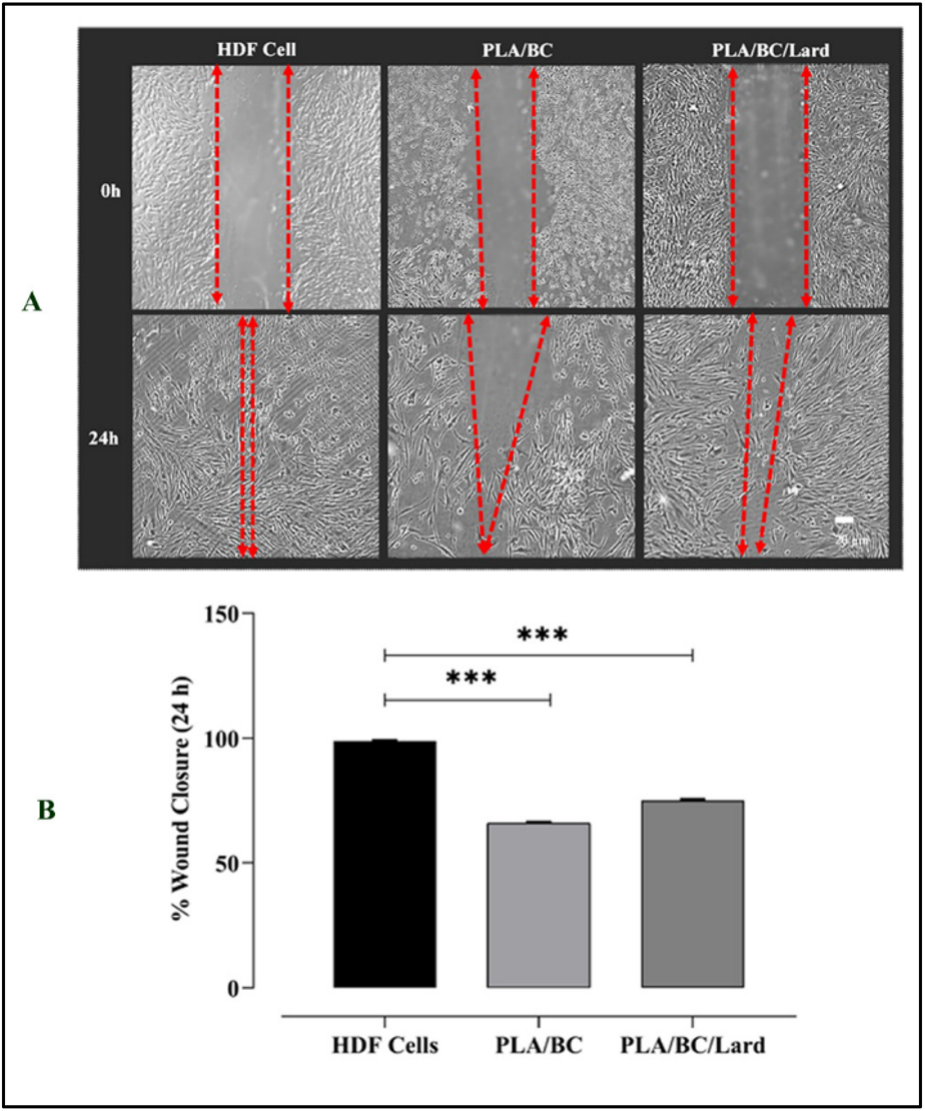
Wound healing capacity of HDF cells in the presence of PLA/BC and PLA/BC/lard nanofibers compared to untreated control. (A) Representative images of HDF cell monolayers at 0 h and 24 h after scratch. (B) Quantitative analysis of wound closure at 24 h. Images were analyzed using ImageJ software, and the percentage of wound closure was calculated according to the standard formula. Data are presented as mean ± SEM (*n* = 3). Statistical analysis was performed using one-way ANOVA followed by Tukey's multiple comparisons test. *p < 0.05, **p < 0.01, ***p < 0.001; ns, not significant (*p* > 0.05).

Wound closure trends in the PLA/BC and PLA/BC/lard groups in HDF cells, together with the untreated control, are presented in Fig. 9. The PLA/BC/lard group showed higher wound closure (∼75%) and greater migration-associated closure than the PLA/BC group (∼65%) (***p < 0.001, Fig. 9B). The untreated group was used as a reference condition for comparative interpretation. Representative scratch images (0 h and 24 h) and quantitative closure analysis are shown in Fig. 9A and B, respectively.

#### 3.6.3. Cytokine response of HDF cells on nanofibers

**Fig. 10:**
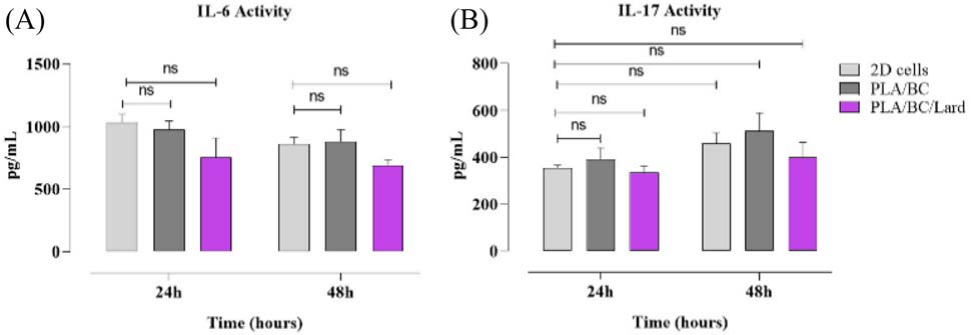
Cytokine response of HDF cells on nanofibers. The secretion levels of IL-6 (A) and IL-17 (B) were quantified using ELISA kits (BT-Lab, Hangzhou, China) according to the manufacturer's instructions. Data are presented as mean ± SEM (*n* = 3). Statistical analysis was performed using one-way ANOVA followed by Tukey's multiple comparisons test. *p < 0.05, **p < 0.01, ***p < 0.001; ns, not significant (*p* > 0.05).

To evaluate cytokine response, IL-6 and IL-17 secretion levels were measured by ELISA at 24 h and 48 h following treatment with PLA/BC and PLA/BC/lard nanofibers. Compared with PLA/BC, PLA/BC/lard showed lower mean cytokine levels at both time points (IL-6: ∼980 *vs.* ∼760 pg mL^−1^ at 24 h; ∼890 *vs.* ∼690 pg mL^−1^ at 48 h; IL-17: ∼400 *vs.* ∼330 pg mL^−1^ at 24 h; ∼520 *vs.* ∼400 pg mL^−1^ at 48 h). However, these differences did not reach statistical significance (*p* > 0.05). Therefore, the cytokine profile is interpreted as a non-significant downward trend at the composite level rather than definitive inhibition by a specific component. In parallel, early wound closure at 24 h was higher for PLA/BC/lard than PLA/BC (∼75% *vs.* ∼65%), indicating a modest functional benefit under *in vitro* conditions (Fig. 10).

Wound healing progresses through overlapping phases (hemostasis, inflammation, proliferation, and remodeling).^64^ Within this framework, the scratch assay is interpreted as a readout of collective migration linked mainly to the proliferative/re-epithelialization component.^65,66^ Therefore, the 24–48 h closure findings indicate early phase-relevant biological behavior, while broader high-inflammatory wound processes should be evaluated in advanced models.^67^

Lard should be considered a mixed lipid matrix containing both saturated and unsaturated fatty acids rather than a single fatty-acid entity.^68^ Because PLA/BC/lard is a multicomponent system, the observed cytokine shifts cannot be causally attributed to a single constituent in the current design. Mechanistically, palmitic-acid-dominant signaling has been associated with pro-inflammatory activation, including TLR/NF-κB-related pathways,^69,70^ whereas oleic-acid-associated signaling has been linked to attenuation of inflammatory signaling in relevant models.^71,72^ In addition, direct phospholipid-bilayer integrity endpoints were not measured in this study; therefore, potential membrane-level risk is interpreted indirectly from short-term cytocompatibility and migration data. Since cytokines were measured under basal conditions (without TNF-α/IL-1β priming), the lower IL-6/IL-17 means are interpreted conservatively as a non-significant trend (*p* > 0.05). Future validation will employ TNF-α/IL-1β-primed inflammatory models with expanded marker panels (TNF-α, IL-10, CD86, CD206) to assess immunomodulatory behavior under burn-relevant conditions. Moreover, IL-17 biology in wound repair is phase- and context-dependent, supporting cautious interpretation.^73^ Dedicated component-resolved and inflammatory-challenge experiments are required to establish causality and refine mechanism-level interpretation.

Building on this, recent advancements have introduced multifunctional nanoplatforms that actively disrupt bacterial biofilms to promote healing in infected wounds.^74^ Similarly, nano-sized coordination polymer particles (CPPs) have been highlighted for their synergistic antibacterial and therapeutic capabilities.^75^ Furthermore, novel ‘attack and defense’ antimicrobial strategies have shown promise in effectively neutralizing pathogens while maintaining biocompatibility.^76^ Collectively, these studies emphasize the shift towards dressings that actively modulate the biological microenvironment, aligning with the immunomodulatory potential observed in our PLA/BC/lard nanofibers.

Burn-wound biology involves a complex and overlapping repair sequence with strong inflammatory and remodeling components.^64^ Within this context, the present dataset (cytocompatibility, migration-associated closure, and basal cytokine profiling) should be interpreted as early *in vitro* evidence of material performance rather than definitive burn-therapeutic efficacy. Under the tested conditions, PLA/BC/lard showed a favorable short-term profile. The next phase of this work will extend validation in burn-relevant high-inflammatory settings. Although antimicrobial assays were performed, no statistically significant antimicrobial activity was detected under the current formulation and test conditions. In addition, no *in vivo* burn model was included in the present study. Therefore, expanded microbiological testing, transepidermal water loss (TEWL)/moisture-retention analysis, inflammatory-challenge models, and *in vivo* burn validation are planned as next-phase studies.

## 4. Conclusions

This study developed PLA/BC/lard nanofibrous composites and showed that incorporating a natural lipid phase can improve the flexibility of PLA-based wound-dressing matrices. BC provided structural support and hydrophilicity, while lard acted as a plasticizing component that increased biomechanical adaptability under the tested conditions. *In vitro* assays demonstrated favorable cytocompatibility and support for fibroblast-associated wound-healing functions. Although IL-6 and IL-17 levels were lower in the PLA/BC/lard group, these differences were not statistically significant (*p* > 0.05) and should therefore be interpreted as a preliminary biological trend. Overall, these findings provide an initial proof-of-concept for a lard-containing lipid–polymer hybrid wound-dressing platform. Further inflammatory-challenge, component-resolved, and *in vivo* burn-wound studies are needed to confirm efficacy and support clinical translation.

Nevertheless, several challenges must be considered when translating these composites to burn wound models. Burn injuries present a highly dynamic and inflammatory microenvironment characterized by elevated cytokine levels, increased exudate, and compromised barrier function. Specifically, the hydrophobic nature of lard observed in swelling tests suggests that managing high volumes of exudate to prevent excessive fluid accumulation at the wound interface will be a key challenge.

Despite the promising findings regarding the mechanical and biological properties of PLA/BC/lard nanofibers, this study has certain limitations. First, the degradation behavior was evaluated over a relatively short period of 5 days (Fig. 5b). While significant mass loss (∼25%) was observed due to the release of lipid components, this timeframe may not fully capture the long-term hydrolytic degradation profile of the PLA matrix, which typically occurs over months. Consequently, short-term tests might underestimate the structural changes and mechanical integrity loss that could occur during prolonged clinical use. Second, the biological assessments were restricted to *in vitro* cell culture models. Although these assays confirmed cytocompatibility and immunomodulatory potential, they cannot fully replicate the complex multicellular and inflammatory environment of a physiological burn wound.

Future work will therefore focus on comprehensive *in vivo* validation using established burn wound models to evaluate healing efficacy, inflammatory modulation, and degradation behavior under physiological conditions. The controlled release and stability of lipid components, along with the balance between occlusivity and breathability, remain critical parameters for further investigation. Further optimization of lipid content and fiber architecture will be pursued to fine-tune mechanical performance towards ‘mechanically active’ dressing concepts and optimize moisture regulation. Together, these efforts aim to advance the PLA/BC/lard system toward clinically relevant, scalable, and patient-friendly wound dressing applications.

## Author contributions

All authors contributed to the study and approved the final version of the manuscript. T. A. and N. I. led the methodology, investigation, formal analysis, and data curation processes. O. K., E. Y., and I. A. contributed to the investigation phase. Z. K. performed formal analysis and visualization. O. G. and C. D. were responsible for conceptualization, methodology design, validation, and visualization. C. D. also managed project administration. B. A. provided supervision and resources alongside O. G. and C. D. regarding the study's infrastructure. T. A., N. I., O. K., E. Y., Z. K., and I. A. wrote the original draft, while T. A., N. I., Z. K., O. G., and C. D. reviewed and edited the manuscript.

## Conflicts of interest

The authors declare no competing financial interest.

## Data Availability

The original contributions presented in the study are included in the article, further inquiries can be directed to the corresponding authors.
